# Infestation by potato tuber moth restructures microbial communities in flue-cured tobacco rhizosphere and non-rhizosphere soils

**DOI:** 10.3389/fpls.2025.1670207

**Published:** 2025-09-24

**Authors:** Ganlin Sun, Zhi Li, Guang Wang, Haosheng Cai, Junkun Yu, Zhengbin Li, Bin Chen, Guanli Xiao

**Affiliations:** ^1^ College of Plant Protection, Yunnan Agricultural University, Kunming, China; ^2^ College of Agronomy and Biotechnology, Yunnan Agricultural University, Kunming, China

**Keywords:** potato tuber moth, *Phthorimaea operculella*, flue-cured tobacco, feeding stress, aboveground-underground interactions, non-rhizosphere microorganisms

## Abstract

**Introduction:**

The rhizosphere microbiota is associated with the plant response to phytophagous pest infestation through the plant-rhizosphere microbe axis. However, the responses of microbial community characteristics of flue-cured tobacco rhizosphere and non-rhizosphere soil to potato tuber moth (PTM) Phthorimaea operculella larval feeding is unclear.

**Methods:**

In this study, the microbial structural composition was analysed in the rhizosphere and non-rhizosphere soil of healthy and PTM infested flue-cured tobacco plants at the vigorous growth stage collected from the field (with four replicates per group) using Illumina MiSeq sequencing. The featured microbes, co-occurrence networks, and potential functions of tobacco rhizosphere and non-rhizosphere soil microbial communities were analysed.

**Results:**

Amplicon data analyses showed that PTM infestation altered the microbial community composition in tobacco rhizosphere and non-rhizosphere and this alteration was similar between these two soil types. PTM infested plants showed enrichment of distinct microbial genera. For instance, the rhizosphere soil showed increased abundances of *Gemmatimonas* (bacteria) and *Humicola* (fungi), while the non-rhizosphere soil was enriched with *Streptomyces* (bacteria) and *Penicillium* (fungi). In contrast, the rhizosphere of healthy plants were characterized by enrichment of *Gaiella* (bacteria) and *Trichoderma*, *Talaromyces* (fungi), along with the non-rhizosphere soil dominated by *Sphingomonas* (bacteria) and *Cordana* (fungi). Furthermore, PTM infestation altered the potential functions of flue-cured tobacco rhizosphere and non-rhizosphere soils, and reduced the complexity of rhizosphere bacterial and fungal communities, as well as the non-rhizosphere fungal community. Notable changes were observed in bacterial metabolic pathways and significantly up-regulated the function of symbiotroph of fungi (Lichenized) (*P* < 0.05).

**Discussion:**

Together, these results enhance our understanding of how the underground microbiome of flue-cured tobacco responds to aboveground phytophagous insect (PTM) infestation, providing valuable insights that could facilitate translation into more effective PTM management practices.

## 1 Introduction

The potato tuber moth (PTM), *Phthorimaea operculella* Zeller (Lepidoptera: Gelechiidae), is a notorious worldwide pest of potato that is active all year in most tropical and subtropical regions (Rondon, 2010; Gao et al., 2024). The damaging stage of the pest is the larva which attacks all vegetative parts of Solanaceae plants, including potato, tobacco, tomato, eggplant, and pepper (Adhikari et al., 2022). In southwestern regions of China, such as Yunnan, Guizhou, and Sichuan Provinces, the PTM not only harms potato, but also damages flue-cured tobacco, leading to quality destruction and economic losses (Gao, 2018; Li et al., 2020). This is mainly due to the overlap in flue-cured tobacco and potato growing season, which provides ample food sources and favorable environmental conditions for PTM development (Yan et al., 2022). However, current research on PTM has focused on its impact on potato, with little attention to its effects on flue-cured tobacco. Given that flue-cured tobacco is an economically important crop susceptible to PTM infestation during its growth, and its cultivation environment favors the proliferation of this pest (Rivera and Burrack, 2012; Yan et al., 2022). It is of significant importance to further understand on the interactions between PTM and flue-cured tobacco.

Microorganisms accompany plants throughout their life cycle, forming a tight plant-microbe associations that are important for physiological activities such as nutrient uptake, metabolism, and immune regulation in plants (Liu et al., 2024; Xu et al., 2025). In particular, rhizosphere soil microorganisms are closely associated with the plant root system and play a dual role when plants experience various biotic (insects and pathogens) and abiotic (heavy metal pollution, drought, floods, and salinity) stresses (Ling et al., 2022; Luo et al., 2022; Ge et al., 2023). For instance, specific beneficial microbiota communities can enhance the resilience and growth of the plants by facilitating nutrient uptake, synthesizing antibiotics, or inducing systemic resistance (Trivedi et al., 2020; Compant et al., 2024). Adverse environmental stresses may disrupt soil microbial homeostasis, intensify nutrient competition or deleterious metabolites, or trigger an abnormal accumulation of potential pathogens, all of which may amplify the negative impacts of stress on plants (Berendsen et al., 2012; Zheng et al., 2022). Understanding the structure and function of relevant microbiome communities under such stress conditions may provide an avenue for the establishment of new resource-efficient and resilient agroecosystems (Singh et al., 2020; Gao et al., 2024).

Infestation by phytophagous insects poses a widespread threat to plants (Divekar et al., 2022). In response to herbivore attack, plants activate defense response characterized by the accumulation of secondary metabolites and inhibitory proteins (Kerchev et al., 2012). Notably, the rhizosphere microorganisms can help plants resist pest feeding (Wang et al., 2022; Xu et al., 2023). Further research has revealed that aboveground phytophagous insects can modify the rhizosphere microbial community by modulating root exudate composition through hormone-mediated signaling pathways such as those involving salicylic acid and Jasmonic acid, thereby feedback influencing plant-insect interaction outcomes (Pineda et al., 2020; Pang et al., 2021; Mostafa et al., 2022). For example, sap-sucking insects (*Bemisia tabaci* and *Macrosiphum euphorbiae*) enhance plant resistance levels in distinct ways by reshaping crop rhizosphere microbial structures. *Bemisia tabaci* infestation promotes the recruitment of beneficial *Pseudomonas* (Kong et al., 2016), while *Macrosiphum euphorbiae* influences the insect resistance performance of subsequent plant generations through soil legacy effects (French et al., 2021). Foliar pest (*Plagiodera versicolora*) infestation enriches insect pathogenic *Pseudomonas*, thereby enhancing willow resistance (Wang et al., 2024). Leaf-mining insect (*Liriomyza trifolii*) infestation induces plants to recruit the nitrogen-fixing bacterium *Bradyrhizobium* as a core rhizosphere microbe, reducing the insect’s fitness (Gao et al., 2024). Similarly, aboveground infestation stress by PTM on potato plants alters the rhizosphere bacterial community structure and promotes the enrichment of the growth-promoting bacterium *Arthrobacter* (Li et al., 2023, 2024). These studies indicate that insects with different feeding habits can primarily drive the community dynamics of rhizosphere associated microbiota. However, the impact of aboveground insect infestation on non-rhizosphere soil microbiomes remains poorly understood. This gap significantly limits our ability to predict the responses of non-rhizosphere soil to insect infestation.

Although non-rhizosphere soil does not directly contact plant fine roots and root exudates, they may respond to aboveground stresses indirectly through changes in soil physicochemical properties affecting community structure and function (Custer et al., 2020; Hou et al., 2021; Yu et al., 2024). Unlike rhizosphere soil, which is directly regulated by plant roots, non-rhizosphere soil lacks such direct influence (Berendsen et al., 2012). This results in generally lower nutrient content (Liu et al., 2022) and microbial biomass (Xu et al., 2025) in non-rhizosphere soil. Due to the absence of a continuous supply of root exudates, the number of active microorganisms in this region tends to be low, posing challenges for analyzing microbial dynamics (Lange et al., 2024). Recent studies have shown distinct responses of microbial communities in rhizosphere and non-rhizosphere soils to plant reactions under biotic and abiotic stresses such as pathogen infection (Hou et al., 2025), climate warming (Yang et al., 2023), and drought stress (Li et al., 2024), highlighting the necessity of systematically comparing microbial responses across different soil niches to plant stress resistance mechanisms. Therefore, understanding the structure and function of microbial communities in the rhizosphere and non-rhizosphere soils of healthy and PTM larvae-infested plants is crucial for developing microbiome-based ecological strategies for pest control.

In the present study, we collected rhizosphere and non-rhizosphere soils from both healthy and PTM larvae-infested flue-cured tobacco plant in the Luoping County, Yunnan, China. Using Illumina MiSeq sequencing, we analyzed differences in the diversity, abundance, and composition of microbial communities between the treatments data. In addition, we compared co-occurrence networks of microbiomes and predicted microbial functional traits. The objectives of this study were to (i) gain new insights into the response of flue-cured tobacco rhizosphere and non-rhizosphere soil microbiomes to PTM larvae infestation, (ii) reveal interactions among phytophagous insect-Plant Soil microbe, and (iii) provide a foundation for the development of integrated pest management strategies based on microbiome regulation.

## 2 Materials and methods

### 2.1 Sample collection

Soil samples from healthy and PTM larvae-infected tobacco were collected separately using the five-point sampling method across three contiguous tobacco fields in July 2024 in the tobacco-planting area of Luoping County, Qujing City, Yunnan Province (25°19′40″N, 104°22′34″E, mean elevation 1955.8 m). Ten plants in total were sampled from each field, comprising five healthy plants and five infected plants (Yao et al., 2024). The soil samples from healthy and PTM larvae-infected plants were thoroughly mixed separately and then divided into four replications (Zhong et al., 2024).Tobacco plants were in the vigorous growth stage (45~55 days after transplanting) and belonged to widely cultivated cv. Yunyan 97. Prior to soil sampling, we confirmed that there were no other plants such as weeds growing near the tobacco plants, and the extent of PTM infestation was assessed by evaluating damage and insects counts. Plants exhibiting feeding damage by five PTM larvae per plant, and with four instars of larvae present in mine holes were considered as PTM stressed. In contrast, plants with intact, smooth leaves and without notches and mine holes were considered as healthy.

After selecting the target tobacco plant, the black film was removed and the topsoil was cleared with a sampling spade. The root system was dug out to a depth of 10–20 cm to collect the soil. Loose soil was gently shaken off, and a thin layer of soil (1–2 mm) adhering the root surface of the tobacco plant using a sterile bristle brush This portion was designated as the rhizosphere soil sample, following the methods of Zhong et al. (2024) and Yao et al. (2024). For each target tobacco plant, a composite bulk soil from the surrounding area, not directly attached to roots, was collected and designated as a non-rhizosphere soil sample (Li et al., 2012). The rhizosphere soil samples from healthy tobacco was designated R_CK, while that from PTM-infected tobacco plants was named R_PTM. Correspondingly, the non-rhizosphere soil samples were named NR_CK for healthy tobacco and NR_PTM for PTM-infected tobacco. All samples were then quickly stored in ice boxes for quick transport back to the laboratory and stored in a freezer at -80°C for soil microbiological analysis.

### 2.2 DNA extraction and MiSeq sequencing

The DNA extraction and Illumina MiSeq sequencing of soil samples were conducted following the methodology of Wang et al. (2024). Rhizosphere and non-rhizosphere microorganism genomic DNA isolation, 16 S rRNA, and ITS gene sequencing were completed with the assistance of Majorbio Co. Ltd., Shanghai. Microbial DNA was isolated from 0.5 g of fresh rhizosphere and non-rhizosphere soil from healthy and PTM larvae infestations using the Qiagen E. Z.N. A.^®^ Soil DNA Kit (Omega Bio-Tek, USA). The bacterial primer 338 F (5′-ACTCCTACGGAGGCAGCAG -3′)/806R (5′- GGACTACHVGGTWTCTAAT -3′) (Lee et al., 2012) and fungal primer ITS1F (5′ CTTGGTCATTTAGAGGAAGTAA-3′)/ITS2R (5′-GCTGCGTTCTTC ATCGATGC-3′) (Degnan and Ochman, 2012) were used to amplify the 16 S rRNA V3-V4 region fragment and ITS1 sequence fragment in the extracted DNA, respectively (Zhang et al., 2024). The PCR product was analysed using 2% agarose gel electrophoresis, purified with the AxyPrep DNA Gel Extraction Kit (AXYGEN, Union, CA) and quantified using QuantiFluor-ST™ (Promega, Wisconsin, USA). Combinatorial amplicon libraries were sequenced on the Illumina MiSeq platform using the TruSeq TM DNA Sample Preparation Kit (Illumina, USA) and following the manufacturer’s guidelines. Raw sequences were filtered and trimmed using FLASH (V. 1.2.11) and the Trimmomatic programme, which includes quality trimming, chimera detection and deletion. SILVA database (V. 138) was used for the sequence alignment of 16 S rRNA gene data, whereas Unite database (V. 8.0) was used for the alignment of ITS gene data (Zhong et al., 2024).

### 2.3 Quality control of sequencing data

To ensure the accuracy and reliability of data analyses, quality control and filtering of raw sequencing reads were first performed using FASTP (V. 0.19.6), bases with end quality values below 20 were removed, a 50 bp sliding window was applied to trim sequences when the average quality within the window fell below 20 and reads shorter than 50 bp or containing N bases were filtered out (Chen et al., 2018). Subsequently, FLASH (V. 1.2.11) was used for sequence merging: paired-end reads were merged into single sequences based on overlapping regions (minimum overlap length of 10 bp), with a maximum mismatch rate of 0.2 allowed in the overlap region, and non-conforming sequences were discarded. Samples were distinguished and sequence orientations were corrected based on barcodes and primers, with no mismatches allowed in barcodes and a maximum of 2 mismatches permitted in primers (Magoč and Salzberg, 2011). Finally, High-quality sequences were then denoised using the DADA2 plugin sequence denoising method in QIIME 2 (V. 2020.2) software to obtain the optimized data after quality control splicing (Liu et al., 2024). DADA2 denoised sequences are commonly referred to as ASV (Amplicon Sequence Variant). The sequencing data of fungi and bacteria were deposited in the Sequence Read Length Archive (SRA) database of NCBI (http://www.ncbi.nlm.nih.gov/sra) (Accession number: PRJNA1226995).

### 2.4 Community diversity and composition analysis

Alpha diversity indices (ACE, Chao 1, Shannon and Simpson indices) and sequencing depth coverage of bacterial and fungal communities in rhizosphere and non-rhizosphere soils of tobacco were calculated using Mothur (V. 1.30.2) (Schloss et al., 2009), and an alpha diversity table (alpha_div.csv) containing the diversity indices for each sample was generated. Since the sample selection function in diversity indices analysis module does not support grouping and thus cannot effectively reflect overall differences between treatment groups, the diversity index table was analyzed using one-way ANOVA in SPSS software (V. 26.0, SPSS Inc., Chicago, IL, USA). Prior to analysis, all data were pretested for normality and homogeneity of variances. Differences were tested for significance using the least significant difference (LSD), with a significant level set at *P*<0.05. For beta diversity analyses, the R software (V. 3.3.1) was performed, and the Vegan v2.5–3 package was used to perform principal coordinate analysis (PCoA) based on Bray-Curtis distance to analyze the differences in bacterial and fungal community structures among the samples. The relative abundance of bacteria and fungi in each sample was plotted using with the ggplot2 package in R software (V. 3.3.1) at the phylum and genus level (top 30), and differences in the relative abundance of microorganisms were analysed at the phylum and genus level for the near-species and non-near-species treatments, respectively. LEfSe analysis (Python 3.x) was used to identify species with significant differences in sample classification (top 6, *P*<0.05). The analysis involved first detecting species abundance variations across groups the nonparametric Kruskal-Wallis (KW) sum-rank test to select statistically significant. These were subsequently subjected to a Wilcoxon rank-sum test to analyze the consistency of differences within subgroup comparisons. Finally, Linear Discriminant Analysis (LDA) was applied to estimate the effect size of each discriminative taxon on group separation (Segata et al., 2011).

### 2.5 Microbial network construction

To describe the interactions between soil bacterial or fungal communities, co-occurrence network patterns at the ASV level of these communities were investigated using one-way correlation network plots (Yu et al., 2024). Co-occurrence networks were constructed in R software (V. 3.3.1) with the psych package (Langfelder and Wgcna, 2008), based on Spearman correlation matrix retaining correlations with an absolute coefficient value ≥ 0.6 and a significance level of *P*<0.05 (Jiao et al., 2022). The resulting correlation data were imported into Gephi software (V. 0.10) for nodes and edges visualization (Bastian et al., 2009). From these networks, fundamental topological properties, including the number of nodes, the number of edges, positive and negative correlations, and modularity were derived. Network stability was assessed through negative or positive correlation ratios and modularity (Newman, 2006; Faust, 2021).

### 2.6 Bacterial and fungal functional analyses

Functional prediction of 16S and ITS amplicon sequencing results was performed using Tax4Fun (V. 0.3.1) and FUNGuild (V. 1.0) software, respectively (Aßhauer et al., 2015; Nguyen et al., 2016). Tax4Fun was used to functionally annotate 16S rRNA gene sequences by converting 16S taxonomic lineages based on the Silva database into prokaryotic taxonomic lineages in the KEGG database (Aßhauer et al., 2015). It was used to obtain metabolic pathway information and pathway abundance at three levels. FUNGuild was used to functionally analyze fungal character sheets to obtain guild data (Nguyen et al., 2016).

## 3 Results

### 3.1 Sequencing data statistics

The number of ASVs in bacteria ranged from 2351 to 3067 in each of the 16 samples tested, and the number of ASVs in fungi ranged from 655 to 839 in each of the samples tested, with sequencing coverage extremely close to 100% in all samples (**Supplementary Table S1**). In addition, the Sobs exponential sparsity curves of these sequences tended to be flat in both bacteria and fungi, suggesting that the sequencing depth was sufficiently deep to reflect the structure of the soil bacterial and fungal communities well (**Supplementary Figure S1**).

### 3.2 The diversity and composition of rhizosphere and non-rhizosphere bacteria after PTM larvae infestation

**Figure 1:**
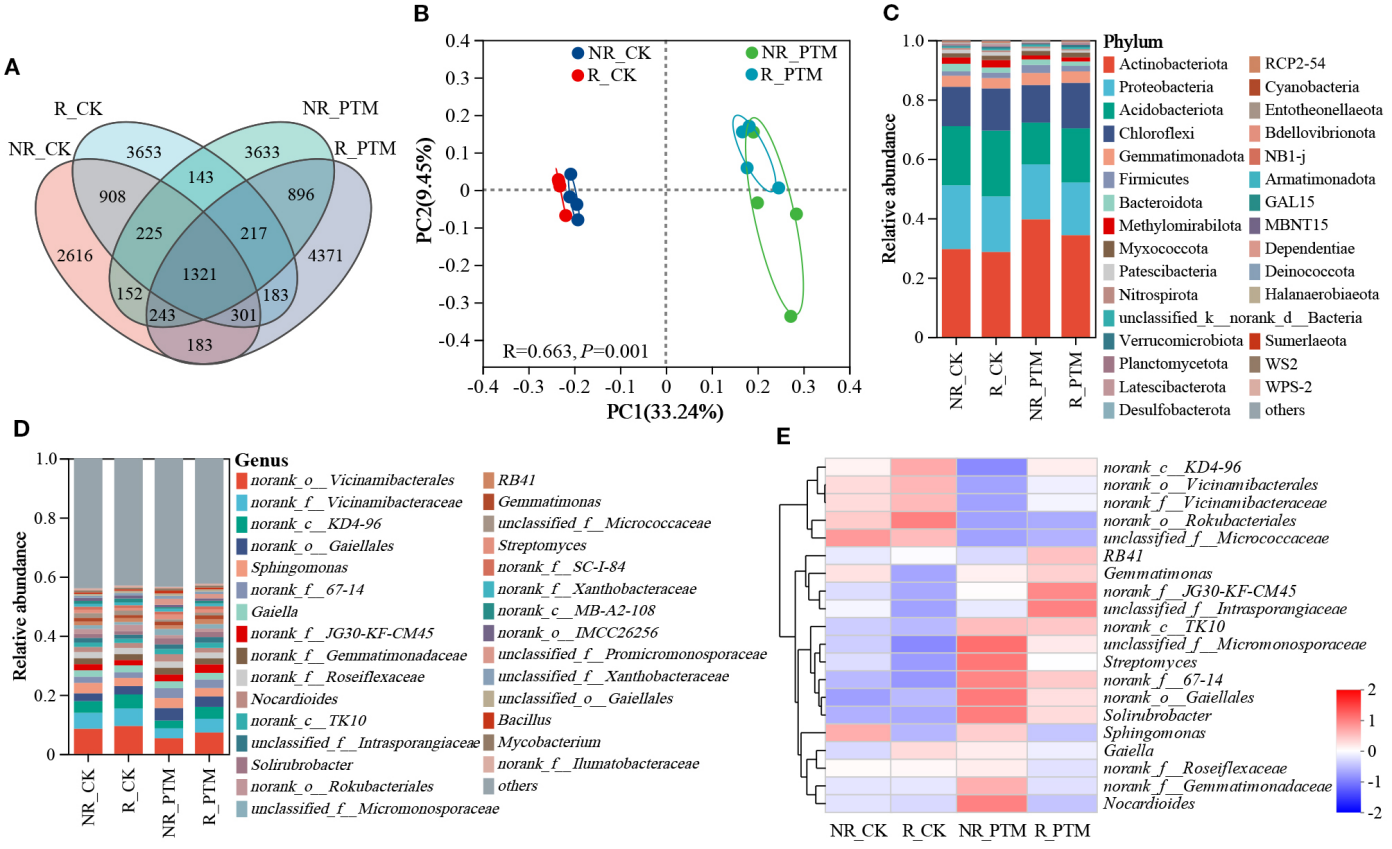
The diversity and composition of bacteria in tobacco rhizosphere and non-rhizosphere soils after by PTM larvae infestation stress. **(A)** Venn diagram of ASVs distribution of bacterial communities. **(B)** PCoA of bacterial communities. **(C)** Phylum-level relative frequency in healthy and PTM larvae-infested tobacco rhizosphere and non-rhizosphere bacteria (top 30). **(D)** Genus-level relative frequency in healthy and PTM larvae-infested tobacco rhizosphere and non-rhizosphere bacteria (top 30). **(E)** Heatmap of genus level of healthy versus PTM larvae-infested tobacco rhizosphere and non-rhizosphere bacteria. “unclassified” in the figure means a scientific name that is not classified in taxonomy; “norank” indicates a scientific name that does not have that level in the classification. PTM, potato tuber moth; NR_CK, non-rhizosphere soil with healthy tobacco; R_CK, rhizosphere soil with healthy tobacco; NR_PTM, non-rhizosphere soil with PTM-infected tobacco; R_PTM, rhizosphere soil with PTM-infected tobacco.

Next, we conducted a comparative analysis of the ASVs to explore the effects of PTM larval infestation stress on tobacco rhizosphere and non-rhizosphere bacterial communities. After clustering of all sample sequences, a total of 19045 ASVs were collected in the bacterial community, of which 1321 were shared ASVs. 6951 and 7715 ASVs were found in the R_CK and R_PTM rhizosphere soils, respectively, while 5949 and 6830 ASVs were found in the NR_CK and NR_PTM non-rhizosphere soils, respectively. Meanwhile, the number of unique ASVs was 2616, 3653, 3633, and 4371 in the groups of NR_CK, R_CK, NR_PTM, and R_PTM, respectively (**Figure 1A**). It indicated that tobacco rhizosphere soil contained higher ASVs of bacteria than non-rhizosphere soil under the same treatments, and that PTM larval infestation increased the ASVs of rhizosphere and non-rhizosphere soil bacteria. The PCoA plot shows a clear separation in bacterial composition between the soils from PTM infested plants and non-infested plants (*P* =0.001, **Figure 1B**). These clusters indicate that attack by PTM has influenced bacterial community structure and caused significant differences in microbial composition. The rhizosphere and non-rhizosphere bacterial communities within the second treatment (non-PTM infested plants) overlap closely, suggesting similar or less distinct impacts on the bacterial communities (**Figure 1B**). In addition, PTM larvae infestation significantly altered bacterial community diversities (Chao and ACE indices, *P <*0.05), but community diversities (Shannon, *P* =0.27, and Simpson indices, *P* =0.52) were not significantly affected (**Table 1**).

**Table 1:** Alpha diversity indices analysis of bacteria and fungi in flue-cured tobacco rhizosphere and non-rhizosphere soils.

Samples	Bacterial			
	ACE	Chao1	Shannon	Simpson
NR_CK	2579.28 ± 92.30b	2570.70 ± 88.53b	7.28 ± 0.02	0.0012 ± 0.00005
R_CK	2832.88 ± 88.33ab	2811.46 ± 81.82ab	7.34 ± 0.01	0.0011 ± 0.00003
NR_PTM	2673.40 ± 75.88ab	2654.56 ± 71.02ab	7.20 ± 0.10	0.0023 ± 0.0011
R_PTM	2984.76 ± 64.06a	2953.06 ± 60.85a	7.35 ± 0.04	0.0016 ± 0.0006
F	4.88	4.9	1.47	0.79
*P*	0.02	0.02	0.27	0.52

	Fungal			
NR CK	778.29 ± 39.78	777.17 ± 39.64	4.86 ± 0.27	0.035 ± 0.021
R_CK	738.10 ± 40.46	736.69 ± 40.39	4.51 ± 0.34	0.062 ± 0.037
NR_PTM	760.26 ± 13.76	759.40 ± 13.47	4.78 ± 0.03	0.033 ± 0.002
R_PTM	783.41 ± 23.71	782.11 ± 23.33	4.76 ± 0.04	0.033 ± 0.002
F	0.42	0.43	0.48	0.44
*P*	0.74	0.74	0.70	0.73

PTM, potato tuber moth; NR_CK, non-rhizosphere soil with healthy tobacco; R_CK, rhizosphere soil with healthy tobacco; NR_PTM, non-rhizosphere soil with PTM-infected tobacco; R_PTM, rhizosphere soil with PTM-infected tobacco. Data in the table are mean ± SE. Different small letters in the same column indicate significant difference at *P <*0.05 level by LSD test.

Illumina MiSeq sequencing results revealed the presence of 38 phyla, 127 classes, 298 orders, 484 genera, and 2154 species of bacteria in all rhizosphere and non-rhizosphere soil samples. The top 30 phyla and genera in abundance were selected to generate column stack diagram of relative species abundance (**Figures 1C, D**). The overall dominant bacterial species in the samples were similar, but the proportion of the same species richness varied between groups. At the phylum level, the bacterial communities were dominated by Actinobacteriota, Proteobacteria, Acidobacteriota, and Chloroflexi. Following PTM larvae infestation, the relative abundance of Actinobacteriota increased by 19.68% and 33.84% in tobacco rhizosphere and non-rhizosphere soils, while the abundance of Acidobacteriota decreased by 17.82% and 29.24%, respectively (**Figure 1C**). At the genus level, PTM larval infestation reduced the proportion of the top 30 phyla in both tobacco rhizosphere and non-rhizosphere soils, from 54.73% (R_CK) to 53.79% (R_PTM) in rhizosphere soils, and from 53.56% (NR_CK) to 51.84% (NR_PTM) in non-rhizosphere soils (**Figure 1D**). Heatmap analysis showed that PTM larval infestation enriched 10 genera in rhizosphere soil (R_PTM), among 8 genera were assigned to known taxa (e.g., *Gemmatimonas*, *Solirubrobacter*, and RB41), whereas two remained unclassified. In non-rhizosphere soil (NR_PTM), 12 genera were enriched, including *Streptomyces*, *Nocardioides* and *Solirubrobacter*, with one genus unclassified. Both values exceeded those of the non-infested plants (six and seven enriched genera in R_CK and NR_CK, respectively) (**Figure 1E**). These results suggest that PTM larval infestation stress altered the bacterial composition of rhizosphere and non-rhizosphere.

### 3.3 The diversity and composition of rhizosphere and non-rhizosphere fungi after PTM larvae infestation

**Figure 2:**
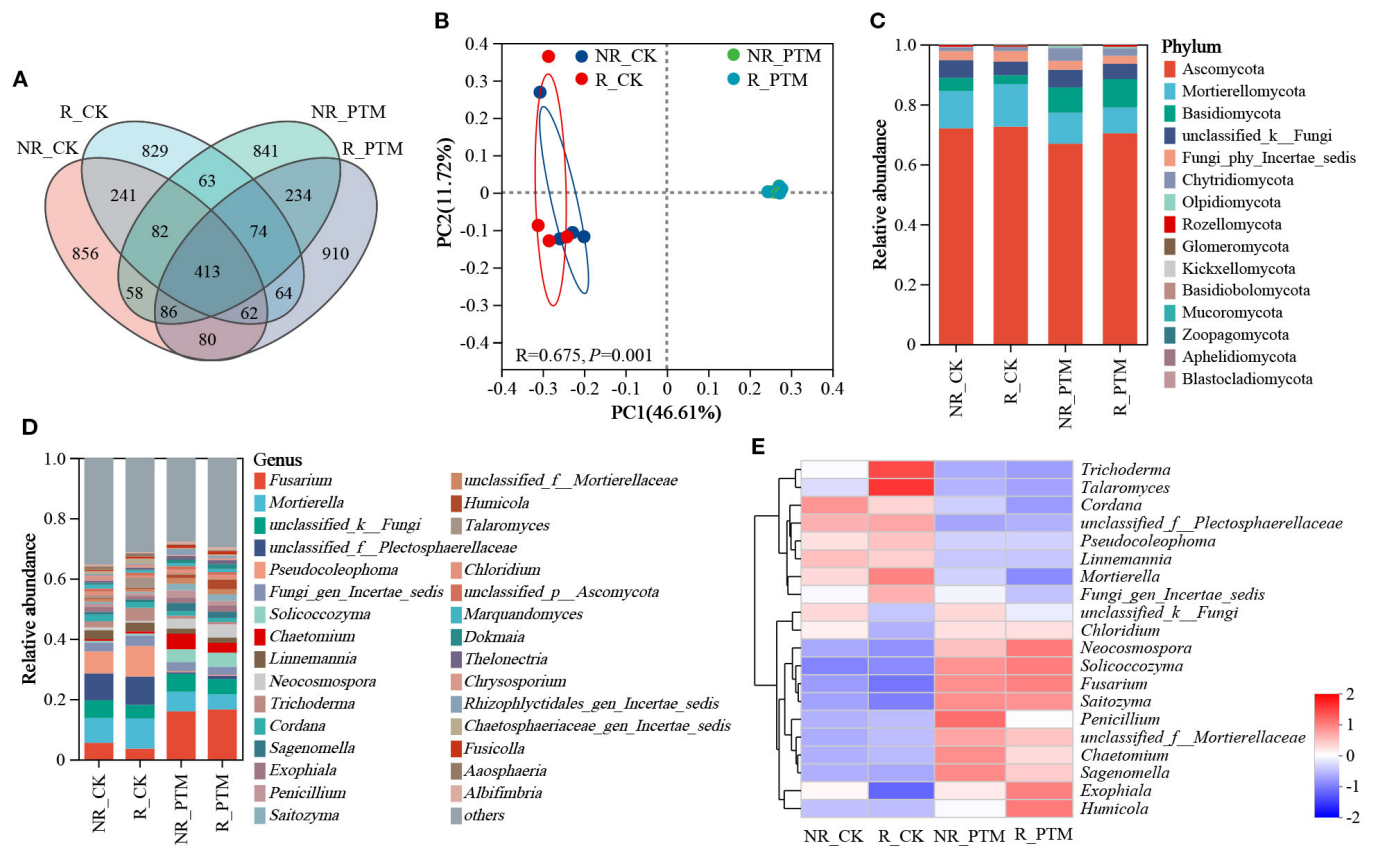
The diversity and composition of fungal in tobacco rhizosphere and non-rhizosphere soils after by PTM larvae infestation. **(A)** Venn diagram of ASVs distribution of fungal communities. **(B)** PCoA of fungal communities. **(C)** Phylum-level relative frequency in healthy and PTM larvae-infested tobacco rhizosphere and non-rhizosphere fungal (top 30). **(D)** Genus-level relative frequency in healthy and PTM larvae-infested tobacco rhizosphere and non-rhizosphere fungal (top 30). **(E)** Heatmap of genus level of healthy versus PTM larvae-infested tobacco rhizosphere and non-rhizosphere fungal. “unclassified” in the figure means a scientific name that is not classified in taxonomy; “norank” indicates a scientific name that does not have that level in the classification. PTM, potato tuber moth; NR_CK, non-rhizosphere soil with healthy tobacco; R_CK, rhizosphere soil with healthy tobacco; NR_PTM, non-rhizosphere soil with PTM-infected tobacco; R_PTM, rhizosphere soil with PTM-infected tobacco.

After sequence clustering, a total of 4893 ASVs were collected from the fungal community, of which 413 were shared ASVs. There were 1878, 1828, 1851, and 1923 ASVs in NR_CK, R_CK, NR_PTM, and R_PTM groups, respectively, and the number of unique ASVs was 856, 829, 841 and 910, respectively (**Figure 2A**). It indicated that PTM larval infestation stress increased the number of rhizosphere soil fungi ASVs but had no significant effect on fungi in non-rhizosphere soil. The PCoA plot shows a clear separation in fungi composition between the soils from PTM infested plants and non-infested plants (*P* =0.001, **Figure 2B**). These clusters indicate that attack by PTM has influenced fungal community structure and caused evident differences in microbial composition. The rhizosphere and non-rhizosphere fungal communities in PTM infected plants showed close overlap, whereas those in non-PTM infected plants remained relatively independent. This indicates that PTM infection exerts differential effects on rhizosphere and non-rhizosphere fungal communities (**Figure 2B**). However, PTM larvae infestation did not significantly alter fungal community diversities (Chao and ACE indices, *P* =0.74) and community diversities (Shannon, *P* =0.70, and Simpson indices, *P* =0.73) (**Table 1**).

A total of 15 phyla, 58 classes, 135 orders, 314 families, 649 genera, and 1051 species of fungi were present in all soil samples in the sequencing results. The overall dominant fungal species in the samples were similar dominated by three phyla Ascomycota, Mortierellomycota, and Basidiomycota (**Figures 2C, D**). Compared to non-PTM infected plants, the rhizosphere and non-rhizosphere soils of PTM infested plants showed a decrease of 3.02% and 7.09% in Ascomycota, a reduction of 39.37% and 17.19% in the abundance of Mortierellomycota, while the abundance of Basidiomycota increased by 219.89% and 91.62%, respectively (**Figure 2C**). At the genus level, the composition of ten most abundant phyla in both rhizosphere and non-rhizosphere soils of PTM infested tobacco changed evidently (**Figure 2D**). The heatmap revealed that, compared to the non-infested plants (both R_CK and NR_CK only enriched with 8 genera), the rhizosphere soil (R_PTM) under PTM larval infestation was enriched with 10 genera. These included *Fusarium*, *Solicoccozyma*, *Chaetomium*, *Neocosmospora*, *Humicala*, *Exophiala*, *Sagenomella*, *Saitozyma*, *Chloridium*, and one unclassified genus. The non-rhizosphere soil (NR_PTM) was enriched with 11 genera, comprising 10 genera shared with R_PTM and two unclassified genera (**Figure 2E**). Overall, PTM larval infestation stress altered the fungal composition in both of tobacco rhizosphere and non-rhizosphere soils of tobacco.

### 3.4 LDA revealed the most characteristic genera of bacteria and fungi in the rhizosphere and non-rhizosphere soil after PTM larvae infestation

**Figure 3:**
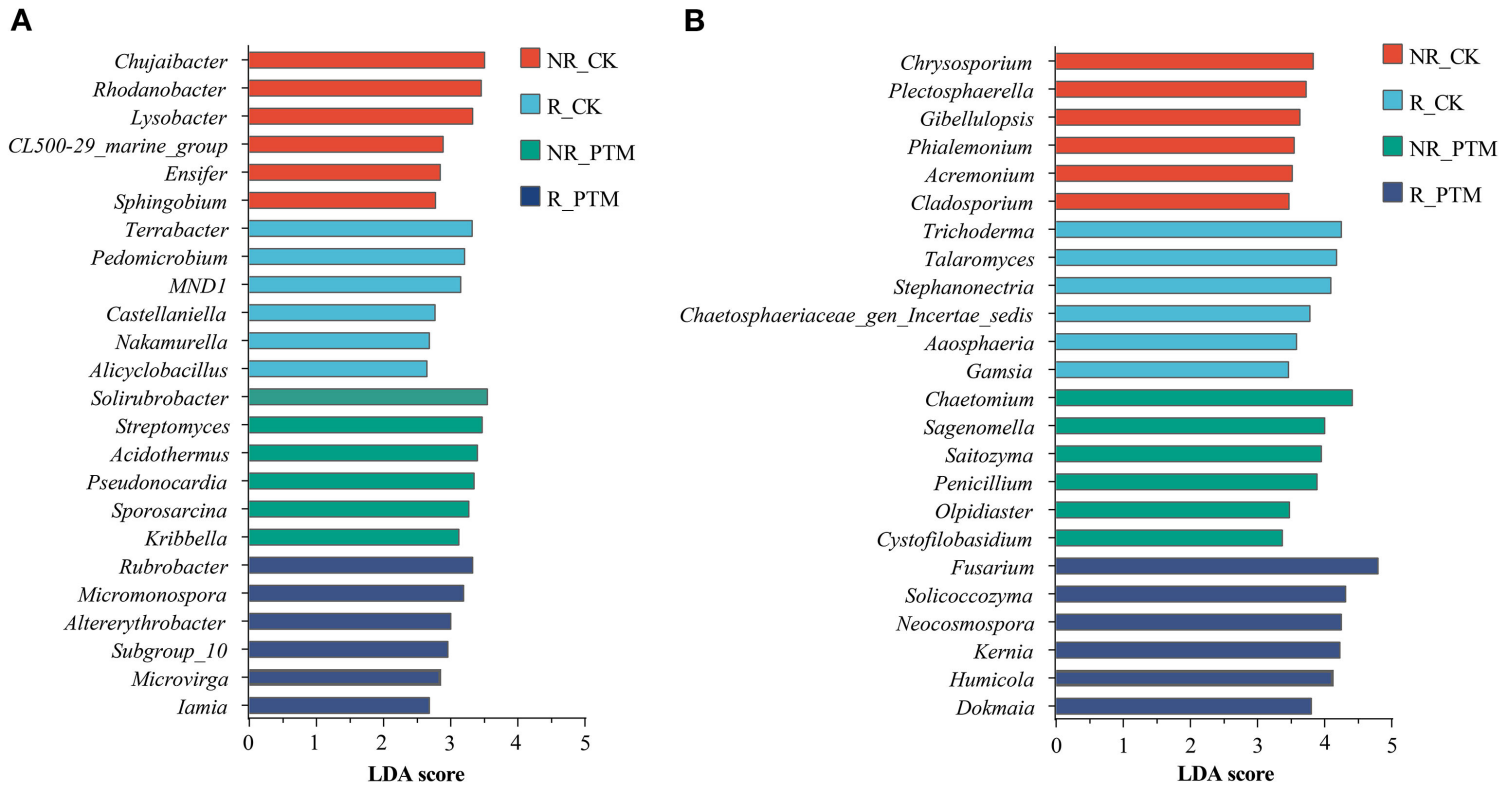
LEfSe analysis of bacteria **(A)** and fungi **(B)** community genus (top 6, *P*<0.05) in tobacco rhizosphere and non-rhizosphere after PTM larvae infestation. PTM, potato tuber moth; NR_CK, non-rhizosphere soil with healthy tobacco; R_CK, rhizosphere soil with healthy tobacco; NR_PTM, non-rhizosphere soil with PTM-infected tobacco; R_PTM, rhizosphere soil with PTM-infected tobacco.

To identify the featured genera associated with stress by PTM larval infestation, we examined microbial abundance profiles at the genus level using the LDA (LEfSe) method. At the bacterial level, LDA values ranged from 2.658 to 3.560 (**Figure 3A**). In non-rhizosphere soils of healthy plants (NR_CK), the most enriched genera was *Chujaibacter*, *Rhodanobacter*, *Lysobacter*, *CL500_29_marine group*, *Ensifer* and *Sphingobium* (*P <*0.05). In the rhizosphere of non-PTM infested plants (R_CK), dominant genera were *Terrabacter*, *Pedomicrobium*, *MNDI*, *Castellaniella*, *Nakamurella*, and *Alicyclobacillus* (*P <*0.05). In non-rhizosphere soils of PTM-infested plants (NR_PTM), the most featured genera were *Solirubrobacter*, *Streptomyces*, *Acidothermus*, *Pseudonocardia*, *Sporosarcina*, and *Kribbella* (*P*<0.05), whereas in the rhizosphere of PTM infested plants (R_PTM), dominant genera were *Rubrobacter*, *Micromonospora*, *Altererythrobacter*, *Subgroup_10*, *Microvirga*, and *Lamia* (*P*<0.05) (**Figure 3A**).

At the fungal level, LDA values ranged from 3.377 to 4.806 (**Figure 3B**). In NR_CK the most featured genera were *Chrysosporium*, *Plectosphaerella*, *Gibellulopsis*, *Phialemonium*, *Acremonium*, and *Cladosporium* (*P*<0.05). In R_CK, the most featured genera were *Trichoderma*, *Talaromyces*, *Stephanonectria*, *Chaetosphaeriaceae_gen_Incertae_sedis*, *Aaosphaeria*, and *Gamsia* (*P*<0.05). In NR_PTM, the most characteristic genera were *Chaetomium*, *Sagenomella*, *Saitozyma*, *Penicillium*, *Olpidiaster*, and *Cystofilobasidium* (*P*<0.05). For R_PTM enriched genera included *Fusarium*, *Solicoccozyma*, *Neocosmospora*, *Kernia*, *Humicola*, and *Dokmaia* (*P*<0.05) (**Figure 3B**).

### 3.5 PTM larval infestation destabilize rhizosphere and non-rhizosphere soil microbial community composition and co-occurrence networks

**Figure 4:**
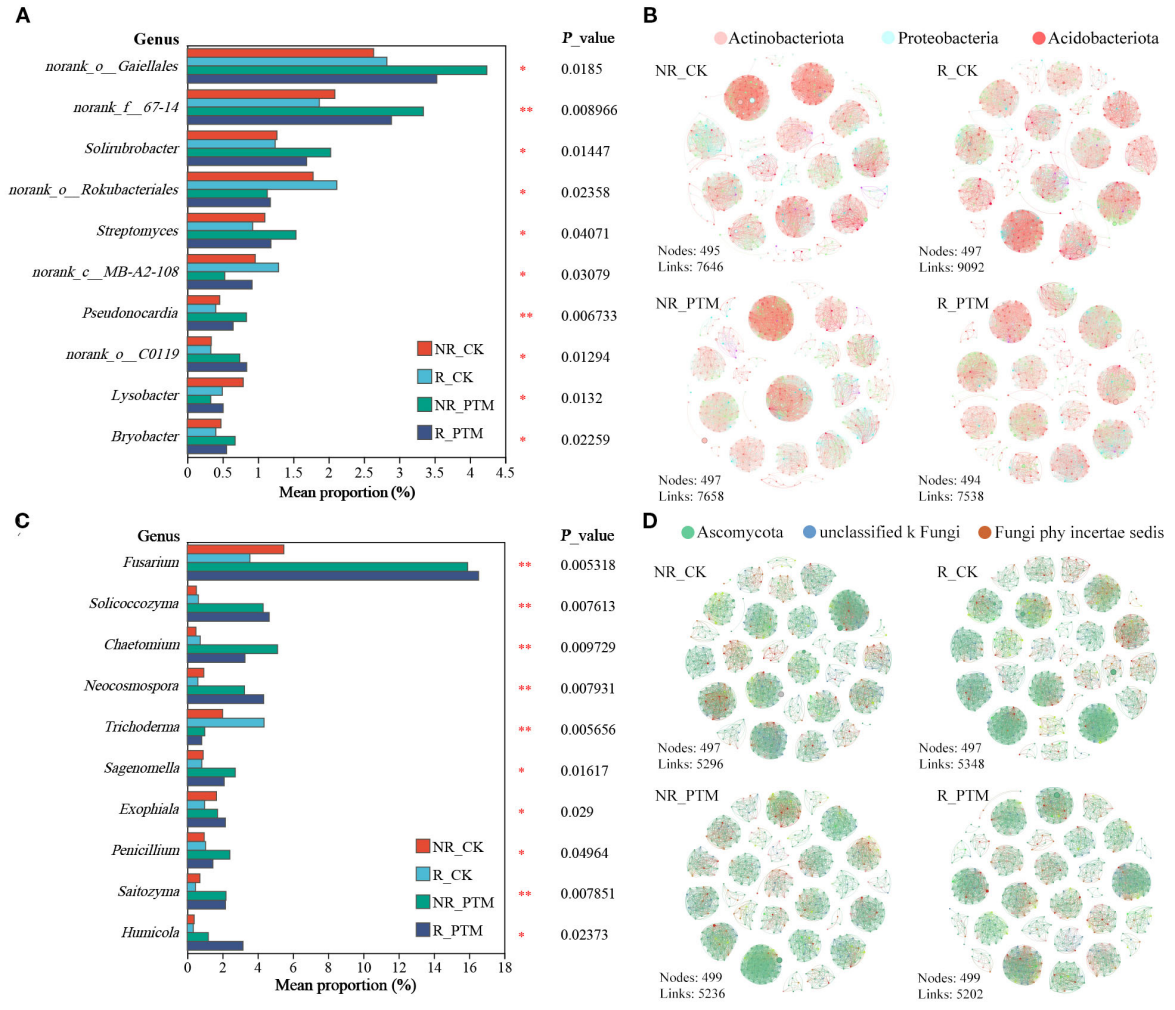
Infestation stress by PTM larvae induced changed tobacco rhizosphere and non-rhizosphere soil microbial community and composition. Histogram showing significantly different bacterial genera **(A)** and fungal genera **(C)**. The co-occurrence networks of PTM larval feeding and healthy tobacco rhizosphere and non-rhizosphere soil bacterial **(B)** and fungal **(D)** communities. PTM, potato tuber moth; NR_CK, non-rhizosphere soil with healthy tobacco; R_CK, rhizosphere soil with healthy tobacco; NR_PTM, non-rhizosphere soil with PTM-infected tobacco; R_PTM, rhizosphere soil with PTM-infected tobacco. ^**^ *P*<0.01, ^*^ *P*<0.05.

To further investigate the effects of PTM larval infestation on tobacco rhizosphere and non-rhizosphere soil microorganisms, we analysed differences in the composition microbial communities between healthy and PTM larval-infested tobacco at the genus level. At the bacterial level, PTM larval infestation led to a significant increase in the abundance of 7 genera in rhizosphere and non-rhizosphere soils (*P <*0.05), including *Solirubrobacter*, *Streptomyces*, *Pseudonocardia* (*P <*0.01), *Bryobacter*, and 3 genera classified under known taxa. Conversely, the abundance of three other genera significantly decreased (*P <*0.05), including *Lysobacter* and 2 genera classified under known taxa (**Figure 4A**). We found that PTM larvae infestation reduced the complexity of the rhizosphere bacterial community and increased the complexity of the non-rhizosphere bacterial community (**Figure 4B**). In healthy tobacco plants, the bacterial community in rhizosphere soil exhibited greater complexity than that in non-rhizosphere soil. This pattern was reversed following PTM larvae infestation, with the non-rhizosphere bacterial community becoming more complex than the rhizosphere. These results suggest that PTM-induced damage reduces the structural complexity of the tobacco rhizosphere bacterial community (**Figure 4B**). Meanwhile, PTM larvae infestation increased the ratio of positive to negative correlation between tobacco non-root and bacterial genera, with the positive correlation increasing from 50.99% (NR_CK) to 59.24% (NR_PTM). In contrast the positive to negative correlation ratio in the tobacco rhizosphere remained unchanged, with a both R_CK and R_PTM exhibiting the stable positive correlation of 51.63%.

At the fungi level, PTM larval infestation significantly increased relative abundance of 9 genera in both rhizosphere and non-rhizosphere soils, including *Fusarium*, *Solicoccozyma*, *Chaetomium*, *Neocosmospora*, *Penicillium*, *Saitozyma* (*P <*0.01), and *Sagenomella*, *Exophiala*, *Humicola* (*P <*0.05), while significantly reducing the abundance of *Trichoderma* (*P <*0.01) (**Figure 4C**). The co-occurrence networks showed that both rhizosphere and non-rhizosphere fungal community complexity was reduced by PTM larvae infestation. Positive to negative correlation ratios of tobacco rhizosphere and non-rhizosphere to bacterial genera were also reduced, from 75.37% (R_CK) to 66.67% (R_PTM) and 67.94% (NR_CK) to 62.24% (NR_PTM). Furthermore, similar to bacterial communities, fungal communities exhibited higher complexity in healthy rhizosphere soils than in non-rhizosphere soils. However, this pattern was reversed following PTM larvae infestation with non-rhizosphere fungal communities more complex than those in rhizosphere soils. These findings suggest that PTM-induced stress reduces the structural complexity of fungal communities in the tobacco rhizosphere (**Figure 4D**).

### 3.6 Rhizosphere and non-rhizosphere microbial community functional prediction after PTM larvae infestation

**Figure 5:**
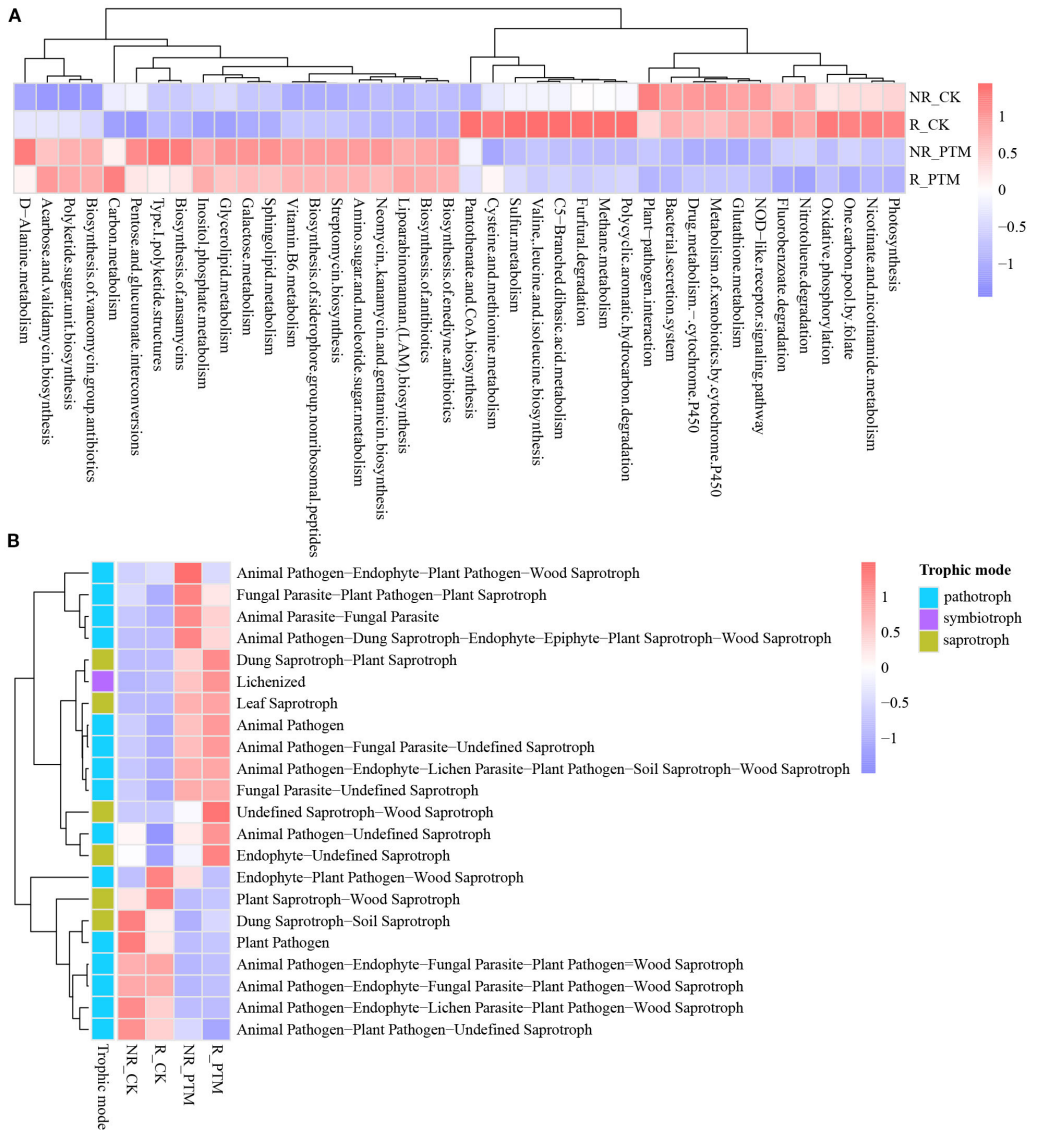
Functional predictions of rhizosphere and non-rhizosphere microbial communities in healthy and PTM larvae-infested tobacco. **(A)** Tax4Fun is a statistically differentiated prediction of (KEGG level 3) bacterial functional traits. **(B)** FUNGuild predicts statistical differences in functional traits for fungi. PTM, potato tuber moth; NR_CK, non-rhizosphere soil with healthy tobacco; R_CK, rhizosphere soil with healthy tobacco; NR_PTM, non-rhizosphere soil with PTM-infected tobacco; R_PTM, rhizosphere soil with PTM-infected tobacco.

Functional analyses of bacteria based on Tax4Fun software predetermined the potential ecological roles of bacterial groups in tobacco rhizosphere and non-rhizosphere soils under both healthy and PTM infested conditions. The analysis of predicted gene functions at the KEGG level 3 identified 60 subfunctions after removing human diseases, undifferentiated and peculiar classifications (**Supplementary Table S2**). The top 40 subfunctions were subjected to functional difference visualization (**Figure 5A**). The predicted functional subcategories showed that rhizosphere and non-rhizosphere samples clustered closely together, whereas healthy samples were more distinct from PTM infested samples. This pattern indicates that PTM larvae infestation significantly altered the function of the soil bacterial community and highlights functional differences between rhizosphere and non-rhizosphere soils. Moreover, metabolic function (92.50%) was the major component. Compared to healthy tobacco, 19 functions were significantly up-regulated and down-regulated in both rhizosphere and non-rhizosphere soils of tobacco infested with PTM larvae. In contrast, Pantothenate and CoA biosynthesis showed significant down-regulation of functions in rhizosphere soil and up-regulation of functions in non-rhizosphere soil. Notably, carbon metabolism was significantly up-regulated only in the rhizosphere soils (**Figure 5A**, **Supplementary Table S2**).

Similarly, functional prediction information was obtained for different fungal samples based on FUNGuild (Fungi Functional Guild) software. After removing human diseases, undifferentiated and peculiar classifications, 22 subfunctions were identified (**Supplementary Table S3**) and functional differences visualized (**Figure 5B**). These subfunctions were classified into three types of trophic modalities: pathotroph, symbiotroph and saprotroph. Of these, pathotroph was the most dominant, accounting for 15 (68.18%). Notably, 7 subfunctions were significantly up-regulated, while 5 were significantly down-regulated in both tobacco rhizosphere and non-rhizosphere soils after infestation by PTM larvae. It is important that of the remaining 3 subfunctions, Animal Pathogen-Undefined Saprotroph was significantly up-regulated only in rhizosphere soils. Conversely, Animal Pathogen-Endophyte-Plant Pathogen-Wood Saprotroph was significantly up-regulated only in non-rhizosphere soils, and Endophyte-Plant Pathogen-Wood Saprotroph showed significant up-regulation in rhizosphere soils and significant down-regulation in non-rhizosphere soils (**Figure 5B**, **Supplementary Table S3**). Among the identified saprotrophs, 6 taxa (27.27%) showed significant changes following PTM larvae infestation, 3 were significantly up-regulated in both rhizosphere and non-rhizosphere soils, 2 were significantly down-regulated, while 1 classified as Endophyte-Undefined Saprotroph was significantly up-regulated only in rhizosphere soils (**Figure 5B**, **Supplementary Table S3**). However, only one symbiotrophic group (Lichenized) was identified and showed significant functional up-regulation in both rhizosphere and non-rhizosphere soils after infestation with PTM larvae (**Figure 5B**, **Supplementary Table S3**). Consequently, the functioning of bacterial and fungal communities after exposure to PTM larvae varies widely, both from rhizosphere and non-rhizosphere soils, and from healthy and PTM larvae-infested conditions.

## 4 Discussion

Aboveground herbivorous insects’ infestation has been demonstrated in multiple studies to alter the composition and performance of plant-associated soil biota (Custer et al., 2020; Gao et al., 2024; Shi et al., 2022; Wang et al., 2024). This study similarly found that PTM infestation significantly altered the microbial composition of both rhizosphere and non-rhizosphere soils in tobacco, subsequently affecting the complexity and potential functional characteristics of the rhizosphere microbiome. These changes may further regulate pest adaptation and plant resistance through tripartite microbe-plant-insect interactions (Pineda et al., 2020; French et al., 2021). Previous studies on PTM have demonstrated that its aboveground infestation behaviour induces physiological responses in different plant parts of potato, including the accumulation of defensive compounds in aboveground leaves and the regulation of related gene expression, thereby enhancing overall plant resistance (Mao et al., 2022; Zhu et al., 2023). Additionally, it leads to a restructuring of rhizosphere bacterial communities with increased beneficial bacterial abundance (Li et al., 2023, 2024).

A growing number of studies suggest that timely plant-mediated restructuring of the rhizosphere microbiota contributes to enhanced plant resilience to both biotic and abiotic stresses (Ling et al., 2022; Ge et al., 2023; Trivedi et al., 2020). In this study, we observed the abundance level of Actinobacteriota (bacteria) and Basidiomycota (fungi) was significantly elevated in both rhizosphere and non-rhizosphere soils of tobacco infested with PTM larvae. These findings are in line with the previous reports showing that PTM-induced stress increased the relative abundance of Actinobacteriota in the potato rhizosphere bacterial community (Li et al., 2023, 2024). Interestingly, Actinobacteriota and Basidiomycota are typical beneficial microorganisms in soil. Actinobacteriota has been developed as an effective biocontrol agent against plant pathogens, and its members contain a large number of plant-promoting functional and pathogen-antagonistic bacteria, which promote plant growth while also inducing plant systemic resistance to inhibit pathogenic microbial infestation (Ebrahimi-Zarandi et al., 2022). Most of the members of Basidiomycota are known for their ability to accelerate rhizosphere nutrient acquisition by establishing ectomycorrhizal associations with a range of host plants to promote their growth as well as to protect against stress (Martin et al., 2016). For example, *Piriformospora indica* is comparable to *Arbuscular Mycorrhizal* (AM) in terms of plant growth-promoting effects (Jisha et al., 2019). Additionally, PTM infestation increased the bacterial abundance of *Gemmatimonas Solicoccozyma*, and *Streptomyces* genera in tobacco rhizosphere soil, known as plant-beneficial microorganisms (Lu et al., 2020; Yang et al., 2023; Carvajal et al., 2024). Studies have shown that *Gemmatimonas* enhances soil health by phosphate solubilization and participation in nitrogen metabolism (Lu et al., 2020). *Solicoccozyma* stimulates IAA synthesis, promoting plant growth (Carvajal et al., 2024). *Streptomyces* supports host health and growth (Worsley et al., 2020) and alleviate abiotic stress through compounds like pteridic acids (Yang et al., 2023). We also found that PTM larval infestation significantly enriched fungal genera including *Chaetomium*, *Exophiala*, *Penicillium*, *Saitozyma*, and *Humicola*, most of which include strains previously shown to be beneficial to plants. For instance, *Chaetomium globosum* CGSR13 (Goswami et al., 2025), *Exophiala* sp. LHL08 (Khan et al., 2011), *Penicillium* spp. GP15-1 (Hossain et al., 2014), *Saitozyma podzolica* S-77 (Das et al., 2023) and *Humicola phialophoroides* (Yang et al., 2014) exhibits functions of antagonizing soilborne phytopathogens or promoting plant growth. Thus, we speculate that tobacco plants may adapt to PTM larval infestation stress by recruiting these beneficial microbiota. It is well established that manipulating beneficial soil microorganisms can induce systemic resistance in plants and affect the performance of aboveground insects, playing a key role in plants defense herbivorous (Rashid and Chung, 2017; Pineda et al., 2020; Gao et al., 2024). However, specific species in this study have not yet been isolated and characterized, and further research needs to focus on the application of these key functional microorganisms.

In this study, we found that PTM larval infestation significantly enriched *Fusarium* and *Neocosmospora* genera in the tobacco rhizosphere and non-rhizosphere soils. Notably, both genera are recognized as potential plant pathogens (Ekwomadu and Mwanza, 2023; Cruz-Luna et al., 2025). In particular, *Fusarium*-induced root rots and wilt are common soil-borne diseases that significantly affecting tobacco cultivation. To date, more than seven *Fusarium* species have been identified as pathogens of tobacco plants, posing a substantial threat to crop health and productivity (Wang et al., 2020; Liu et al., 2024). The enrichment of *Fusarium* and *Neocosmospora* after PTM infestation suggest that PTM larval feeding may increase the risk of disease infection in tobacco plants, highlighting the need for close attention and integrated management strategies. In addition, PTM larval infestation significantly reduced the abundance of several fungus genera, including *Trichoderma*, *Talaromyces*, *Cordana*, *Pseudocoleophoma*, *Linnemannia*, and *Mortierella* known as plant-beneficial microorganisms (Woo et al., 2023; Kharkwal et al., 2024; Vandepol et al., 2022; Du et al., 2024). This suggests that PTM larval infestation can also reduce the abundance of plant-beneficial microorganisms in the soil. However, this study did not determine if there was a close correlation between PTM larval infestation and the development of tobacco diseases caused by *Fusarium* and *Neocosmospora*. Future field surveys should validate the relationship between PTM larval infestation in tobacco plants and the incidence of root rot and wilt diseases caused by *Fusarium* and *Neocosmospora*.

Rhizosphere soil microbes respond to aboveground stresses indirectly through changes in their community structure and function (Hou et al., 2021; Yu et al., 2024). In this study, PTM larval infestation increased multiple functional pathways related to antibiotics production, amino acid, lipid, and carbon metabolism within both rhizosphere and non-rhizosphere soil bacterial communities. Conversely, it reduced pathways associated with plant-pathogen interactions. In additions, PTM larval infestation led to decline in the relative abundance of several plant pathogen fungal guilds, as identified by FUNGuild in both rhizosphere and non-rhizosphere soils. These functional shifts reflect the adaptive responses of soil microbial communities of tobacco to PTM larval feeding.

The stability of microbial communities is essential to ensure ecosystem functioning (Coyte et al., 2015; Jiao et al., 2022). Previous studies have shown that soil bacterial networks are less stable than fungal networks under biotic and abiotic stresses (De Vries et al., 2018; Custer et al., 2020; Wang et al., 2024). Consistent with previous findings, this study demonstrated that PTM larval infestation induced more pronounced changes in the bacterial than the fungal community, with the rhizosphere microbiome exhibiting greater shifts compared to non-rhizosphere microbiome. This may be due to the slower succession and more consistent composition of fungi (Jin et al., 2024). PTM larval feeding increased the fungi abundance of two genera of plant pathogenic fungi and five genera of beneficial fungi in tobacco rhizosphere soil, while reducing the abundance of four other beneficial fungal genera. These shifts suggest a transformation in the composition and functional potential of the rhizosphere fungal community in response to PTM infestation. In addition, the rhizosphere recognized as a hotspot for plant-soil microbial interactions, demonstrated higher sensitivity to changes in plant physiological status, in contrast to non-rhizosphere soil microbial communities, which are less directly affected by plant root exudates (Berendsen et al., 2012; Lange et al., 2024).

Although this study revealed that PTM larval infestation causes alterations in the composition and potential function of tobacco rhizosphere and non-rhizosphere soils, some limitations remain. Firstly, the findings are solely on Illumina MiSeq sequencing, future research could integrate metagenomics and metabolomics to further validate the expression of functional genes and changes in metabolic products. Secondly, the sampling time and geographical scope were relatively limited, and the lack of soil physicochemical property data (e.g., pH, organic matter content, and moisture) restricted the ability to capture how seasonal variations and environmental factors influence microbial community dynamics. Thirdly, the correlation between the enrichment of the potential pathogen (particularly *Fusarium* spp.) and the occurrence of PTM requires validation through additional field trials. Furthermore, the study lacked controls for potential confounding factors, such as the effects of environmental fluctuations, interactions with other pests, and agronomic practices which may influence microbial community dynamic. Thus, a long-term multi-site experiments are necessary to account for these variables. Finally, the long-term effects of microbial community changes on tobacco growth and yield sld be further explored, which is essential for developing integrated pest control strategies based on microbiome regulation.

## 5 Conclusions

This study showed that PTM larval infestation significantly altered the diversity, composition and function of tobacco rhizosphere and non-rhizosphere microbial communities compared to no infested plants. Notably, the nature of these alterations was largely consistent between rhizosphere and non-rhizosphere soils. In PTM larvae-infested tobacco rhizosphere and non-rhizosphere soils, the beneficial genera *Gemmatimonas*, *Streptomyces*, *Chaetomium*, *Exophiala*, *Penicillium*, *Saitozyma*, and *Humicola* were significantly recruited. However, the abundance of the potentially pathogenic fungi *Fusarium* and *Neocosmospora* was significantly increased. These findings provide new insights into the understanding of the complex interactions among plants, phytophagous insects and soil, which can help us to develop field management strategies for PTM control.

## Data Availability

The datasets presented in this study are publicly available. This data can be found here: https://www.ncbi.nlm.nih.gov, accession number PRJNA1226995.
