# Supplementary material for: Infestation by potato tuber moth restructures microbial communities in flue-cured tobacco rhizosphere and non-rhizosphere soils
Source: Front Plant Sci. 2025 Sep 24;16:1670207. doi: 10.3389/fpls.2025.1670207 (PMC12504494; doi:10.3389/fpls.2025.1670207)
Supplement: Supplementary file 1 [file Supplementaryfile1.docx]

Supplementary Material

List of supporting information:

# Supplementary Figures 1 Bacterial (A) and fungal (B) dilution curves for flue-cured tobacco rhizobacteria and non-rhizobacteria in samples infested by PTM larvae.

**Supplementary Table 1:** Statistics of effective sequencing data of bacteria and fungi.

**Supplementary Table 2:** Statistical differences in predicated bacterial functional characteristics at KEGG level 3 (XLSX).

**Supplementary Table 3:** Statistical differences in predicated fungi functional characteristics at KEGG level 3 (XLSX).

## Supplementary Figures


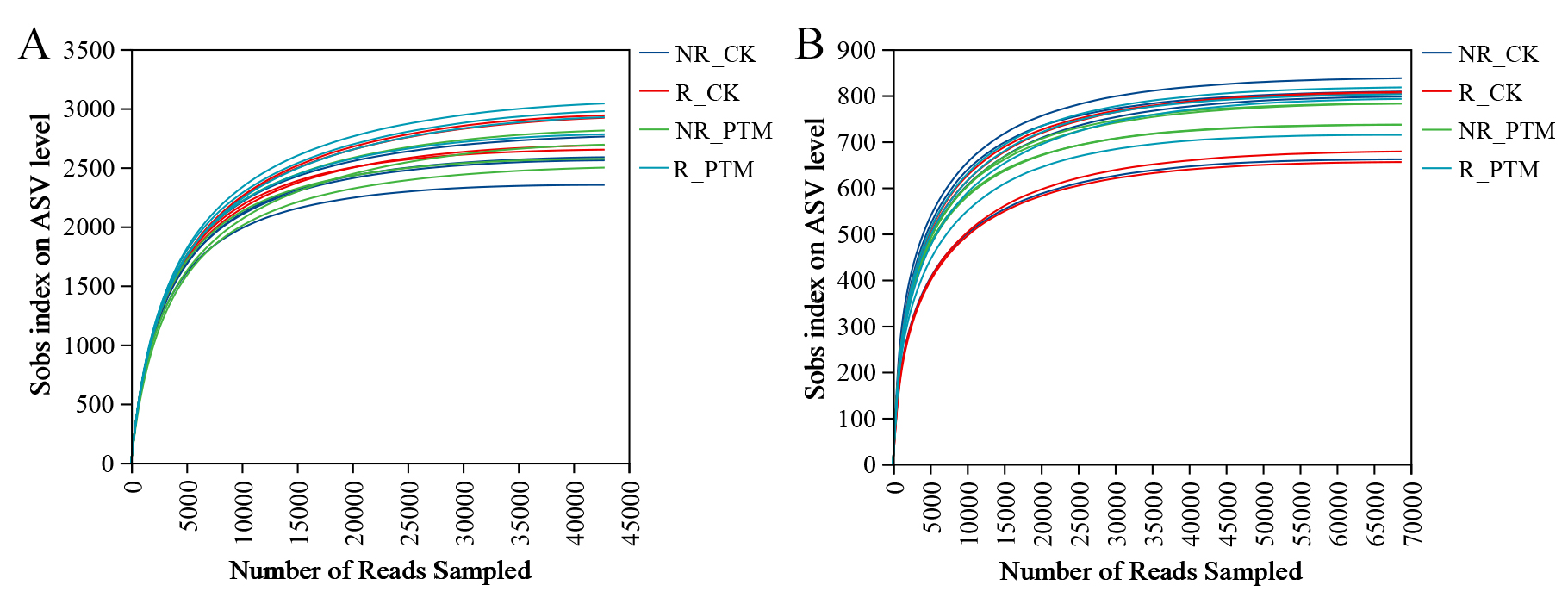


**Fig. S1** Bacterial (A) and fungal (B) dilution curves for flue-cured tobacco rhizobacteria and non-rhizobacteria in samples infested by PTM larvae. ASV, Amplicon Sequence Variant; PTM, potato tuber moth; NR_CK, non-rhizosphere soil with healthy tobacco; R_CK, rhizosphere soil with healthy tobacco; NR_PTM, non-rhizosphere soil with PTM-infected tobacco; R_PTM, rhizosphere soil with PTM-infected flue-cured tobacco

## Supplementary Tables

**Table S1**.Statistics of effective sequencing data of bacteria and fungi

| Samples | Bacteria | |  | Fungi | |
| --- | --- | --- | --- | --- | --- |
|  | ASVs numbers | Coverage |  | ASVs numbers | Coverage |
| NR_CK | 2568.25±86.39 | 0.9989±0.00061 |  | 776.25±39.43 | 0.9998±0.00004 |
| R_CK | 2807.50±80.27 | 0.9978±0.00068 |  | 736.25±40.55 | 0.9999±0.00002 |
| NR_PTM | 2649.00±68.10 | 0.9980±0.00054 |  | 759.00±13.28 | 0.9999±0.00003 |
| R_PTM | 2944.25±59.37 | 0.9967±0.00047 |  | 781.75±23.08 | 0.9999±0.00004 |

Note: ASV, Amplicon Sequence Variant; PTM, potato tuber moth; NR_CK, non-rhizosphere soil with healthy flue-cured tobacco; R_CK, rhizosphere soil with healthy tobacco; NR_PTM, non-rhizosphere soil with PTM-infected flue-cured tobacco; R_PTM, rhizosphere soil with PTM-infected flue-cured tobacco.

**Supplementary Table 2:** Statistical differences in predicated bacterial functional characteristics at KEGG level 3 (XLSX).

**Supplementary Table 3:** Statistical differences in predicated fungi functional characteristics at KEGG level 3 (XLSX).
